# A unified framework for calibration, decoding and resource-aware processing in hybrid quantum–classical machine learning

**DOI:** 10.1098/rsos.260812

**Published:** 2026-10-21

**Authors:** Tunjay Akbarli

**Affiliations:** Omnira, Baku, Azerbaijan

**Keywords:** noisy intermediate-scale quantum computing, hybrid quantum–classical machine learning, quantum error correction, surface-code syndrome decoding, quantum processor calibration, variational quantum eigensolver, observable-driven compression, resource-aware scheduling

## Abstract

Hybrid quantum–classical machine learning on noisy intermediate-scale quantum (NISQ) devices suffers from three coupled overheads: hardware miscalibration, classical syndrome-decoding latency and shot noise on gradient estimators. We present quantum calibration and observable processing (QCOP), a unified closed-loop control layer integrating a spectral calibration engine (SCE), a hierarchical syndrome decoder (HSD) and an observable compression layer (OCL) within a single GPU-accelerated pipeline. Three theoretical bounds characterize the joint effect on logical error rate (LER), convergence rate and observable variance. On device-calibrated noise-model simulators (n=20 seeds), QCOP achieves up to 2× wall-clock training speed-up and approximately 40% variational quantum eigensolver (VQE) energy error reduction relative to the unaugmented baseline. A hardware pilot on *ibm_nairobi* at surface-code distance d=5 (2000 syndrome volumes, 8192 shots each, single calibration window) yields a 1.6× latency reduction and 1.9× LER reduction for the HSD over minimum-weight perfect matching (MWPM)—directionally consistent with simulation but quantitatively smaller, indicating noise sources beyond the model. This pilot exercises the HSD alone: the SCE and the OCL have not been run on physical hardware, and the three modules have never been executed jointly on a device. The end-to-end QCOP benefits—speed-up, energy error reduction and variance reduction—are therefore not hardware-validated, and end-to-end hardware validation of all three modules remains future work. Code and model weights are available at https://github.com/hekimoghlu/QCOP.

## 1. Introduction

Quantum computing in the noisy intermediate-scale quantum (NISQ) era [1] offers processors with tens to hundreds of physical qubits but with two-qubit gate error rates of $10^{-3}$–$10^{-2}$ and coherence times that restrict circuit depth. Hybrid quantum–classical algorithms—which alternate parametric quantum circuits with classical optimizers—are the primary near-term vehicle for exploring quantum advantage in chemistry, combinatorial optimization and supervised learning [2,3]. The practical performance of these algorithms is degraded by three compounding overheads.

First, qubit resonance frequencies drift continuously due to substrate noise, temperature fluctuations and two-level system interactions, shifting effective gate fidelities between calibration cycles [4]. Second, quantum error-correction implementations require syndrome decoding at a rate commensurate with circuit execution; minimum-weight perfect matching (MWPM) [5], the standard classical decoder, runs in $O(d^{3}\log d)$ time per syndrome cycle for surface codes of distance d and becomes a wall-clock bottleneck at d≥7. Third, gradient estimation via parameter-shift rules is shot-noise limited, and this noise is amplified under device miscalibration, increasing the number of circuit evaluations required to reach a target precision.

These three overheads interact: uncorrected drift corrupts observable estimates that decoding alone would otherwise improve, and residual decoding errors inflate the effective shot-noise floor seen by the optimizer. Despite this coupling, calibration, decoding and gradient estimation are typically treated as independent offline steps. The main contribution of this work is the design and simulation-based evaluation of a unified closed-loop execution pipeline, quantum calibration and observable processing (QCOP), that couples real-time spectral calibration, volumetric syndrome decoding and observable compression within a single execution loop, together with lightweight static circuit analysis for crosstalk-aware fragment selection. The individual component techniques are not claimed as novel; the practical advance is their co-design, systematic evaluation under device-calibrated noise models and the theoretical framework quantifying their joint effect. A preliminary hardware pilot validates the decoding module at small code distance; end-to-end hardware validation remains future work and constitutes the principal open question for the framework's practical adoption.

### 1.1. Related work

*Calibration.* Automated qubit calibration has been studied through model-based Bayesian optimization [6] and reinforcement learning [7]. Error mitigation via zero-noise extrapolation (ZNE) [8] and probabilistic error cancellation [9] adds circuit overhead without correcting the underlying pulse schedules. Continuous drift tracking using machine learning has been explored in single-qubit settings [10], but multi-qubit resonance-map-based correction without full calibration sweeps remains less studied.

*Syndrome decoding.* Classical MWPM [5] achieves near-threshold logical error rates (LERs) but cannot sustain real-time operation at large code distances. Neural network decoders have improved LERs and latency over MWPM for surface codes using convolutional architectures [11–13] and, more recently, transformer-based and recurrent models [14,15]. We integrate a volumetric decoder that processes the full d×d×T syndrome tensor jointly, capturing spatiotemporal correlations that earlier spatial-only or separate temporal-aggregation decoders handle less directly.

*Observable post-processing.* Purification-based error mitigation [16], echo sequences [17] and digital ZNE [9] reduce effective noise without direct calibration. Sample-complexity reduction through observable grouping [18] and classical shadows [19] are established methods. The observable compression layer (OCL) in QCOP is trained end-to-end against the hybrid optimization objective, jointly with a shot-noise penalty, rather than applied as a separate pre-processing step.

*Resource scheduling.* Crosstalk-aware scheduling using static circuit analysis for bin-packing on heavy-hex device topologies has been studied in the quantum operating systems literature [20,21]. We incorporate a lightweight version of such analysis for crosstalk-sensitive fragment selection, building on established static circuit analysis methods for crosstalk mitigation.

### 1.2. Summary of contributions

We make the following contributions. First, we present QCOP, a closed-loop control layer that couples spectral calibration, volumetric syndrome decoding and observable compression in a single GPU-accelerated hybrid execution loop; the three-module co-design and its synergistic effect constitute the central contribution, evaluated on device-calibrated simulators and partially validated on physical hardware. Second, we derive the decoding efficiency bound (theorem 2.1), the convergence acceleration bound (theorem 2.2) and the joint observable variance bound (theorem 2.3) with a sample-complexity corollary, all derived under stated assumptions whose limitations are discussed explicitly. Third, we evaluate QCOP on device-calibrated noise models derived from published characterization data for three backend technologies, without device-specific fine-tuning, using n=20 independent random seeds throughout; an ablation study with Wilcoxon significance tests and hyperparameter sensitivity analysis quantify individual module contributions. Fourth, we report a hardware pilot on a five-qubit IBM superconducting processor that provides initial, partial validation of the syndrome-decoding module at d=5.

## 2. Framework

### 2.1. Architecture overview

The QCOP control loop proceeds in five stages per hybrid iteration: (i) the spectral calibration engine (SCE) checks for qubit frequency drift and issues pulse-schedule corrections if needed; (ii) the parametric quantum circuit is executed on the backend; (iii) the hierarchical syndrome decoder (HSD) decodes error syndromes from the syndrome measurement volume and applies a soft correction to raw measurement counts; (iv) the OCL maps the mitigated observable vector to a low-dimensional representation and evaluates the hybrid loss; (v) the classical optimizer updates circuit and compression parameters. An optional static circuit analyser (SCA) runs once per circuit family, identifying qubit pairs with elevated crosstalk exposure for fragment-to-qubit mapping.

Throughout this paper—in the running text, in every table and in every figure and figure caption—the framework is called QCOP, and its components are called the SCE, the HSD, the OCL and the SCA. No alternative name is used for the framework or for any of its modules.

### 2.2. Spectral calibration engine

The SCE monitors each qubit's effective resonance frequency using short diagnostic pulse sequences interleaved with idle circuit slots, producing a two-dimensional (2D) heat map of frequency deviations without requiring a full calibration sweep.

Let $\omega_{i}(t)$ denote the measured resonance frequency of qubit i at time t and $\omega_{i}^{\left(0\right)}$ the reference from the last manual calibration. The per-qubit drift is

(2.1) $$\Delta_{i}(t)=|\omega_{i}(t)-\omega_{i}^{\left(0\right)}|,$$

and a pulse-schedule correction is triggered whenever the root-mean-square drift $\Delta_{\mathrm{rms}}(t)=(n^{-1}\sum_{i}\Delta_{i}(t{)}^{2}{)}^{1/2}$ exceeds a configurable threshold $\delta_{\mathrm{cal}}$ (set to 50kHz in all experiments, corresponding to approximately 0.5% of a typical transmon anharmonicity). The correction is computed by a compact residual network with a cross-attention module that fuses resonance-map features with topology-aware positional embeddings derived from the device connectivity graph. The model was trained on paired (resonance map, correction vector) data generated by sampling from the drift noise model of Burnett *et al.* [4], parametrized by the published 1/f noise spectral density for superconducting devices and equivalent thermal-noise characterizations for trapped-ion and photonic platforms.

### 2.3. Hierarchical syndrome decoder

The HSD processes the syndrome tensor $S\in \{0,1{\}}^{d\times d\times T}$ produced by T measurement rounds on a distance-d surface code. It applies volumetric operations over the spatial patch dimensions to extract per-stabilizer features, then aggregates across the temporal dimension to capture correlated errors spanning multiple rounds—including leakage and measurement hysteresis errors that MWPM treats as independent. The output is a soft-decision correction mask $\hat{c}\in [0,1{]}^{d\times d}$ with a scalar confidence score. The decoder is trained on synthetic syndrome volumes generated under a circuit-level depolarizing noise model with physical error rates $p_{\mathrm{phys}}\in [10^{-4},10^{-2}]$, measurement error rates and leakage contributions drawn from published surface-code characterization experiments [22,23]. This broad training corpus is what enables cross-platform operation without device-specific fine-tuning; this generalization is evaluated empirically in §3.8.

### 2.4. Observable compression layer

The mitigated observable vector $o\in R^{M}$ is mapped to $z=g_{\varphi }(o)\in R^{K}$ (K≪M) by a trainable projection $g_{\varphi }$ learned jointly with the circuit parameters. The projection preserves the dimensions of o most informative for the task loss while discarding shot-noise-dominated components. This is related in spirit to classical shadows [19] and observable grouping [18], differing in being adapted end-to-end to the hybrid optimization objective.

### 2.5. Static circuit analyser

The SCA is an optional module that performs lightweight crosstalk-sensitivity analysis on the device's heavy-hex connectivity graph, building on established static circuit analysis methods for crosstalk mitigation [20,21]. Given a circuit's qubit count n, depth $d_{c}$ and gate count g, it identifies pairs of simultaneously active two-qubit gates whose physical qubits fall within a crosstalk separation threshold $\delta_{\mathrm{sep}}=2$, a value motivated by empirical characterizations of heavy-hex crosstalk profiles [20]. These pairs are flagged as separation-constrained: the co-execution scheduler does not assign circuits with conflicting gate pairs to overlapping time slots. In the single-circuit experiments of this paper, the SCA is used only for qubit-mapping selection; its use for multi-circuit co-execution is discussed in §4.4.

### 2.6. Hybrid loss function

Let $\theta \in R^{p}$ parametrize the quantum circuit and $\varphi \in R^{q}$ the compression projection $g_{\varphi }$. The hybrid loss is

(2.2) $$L_{\mathrm{hyb}}(\theta ,\varphi )=\alpha \;L_{Q}(\theta )+(1-\alpha )\;L_{C}(\varphi )+\mu \;\Omega_{\mathrm{obs}}(\theta ,\varphi ),$$

where α∈(0,1) balances the quantum task loss $L_{Q}$ against the classical network loss $L_{C}$, and

(2.3) $$\Omega_{\mathrm{obs}}(\theta ,\varphi )=\frac{1}{M}\sum_{i=1}^{M}\|\langle O_{i}\rangle_{\;\theta }-g_{\varphi }(x_{i}){\|}^{2}+\nu \;R_{\mathrm{shot}}(\theta ),$$

is the observable compression regularizer. Here, $\langle O_{i}\rangle_{\;\theta }$ is the decoded expectation of observable $O_{i}$, $g_{\varphi }(x_{i})$ is the network's prediction of the same quantity from classical features $x_{i}$ and

(2.4) $$R_{\mathrm{shot}}(\theta )=\sum_{k=1}^{p}{\left(\frac{\partial \langle O\rangle_{\theta }}{\partial \theta_{k}}\right)}^{2}\sigma_{\mathrm{shot}}^{2}(\theta_{k})$$

penalizes parameters whose two-point gradient estimators have high shot-induced variance; $\sigma_{\mathrm{shot}}^{2}(\theta_{k})$ is estimated from repeated evaluations.

### 2.7. Implementation

Listing 1 shows the QCOP execution loop. Complete source code and trained weights are available at github.com/hekimoghlu/QCOP and huggingface.co/thehekimoghlu/QCOP.

**Listing 1. QCOP hybrid optimization loop. Source code is available at github.com/hekimoghlu/QCOP.**

**Figure:**
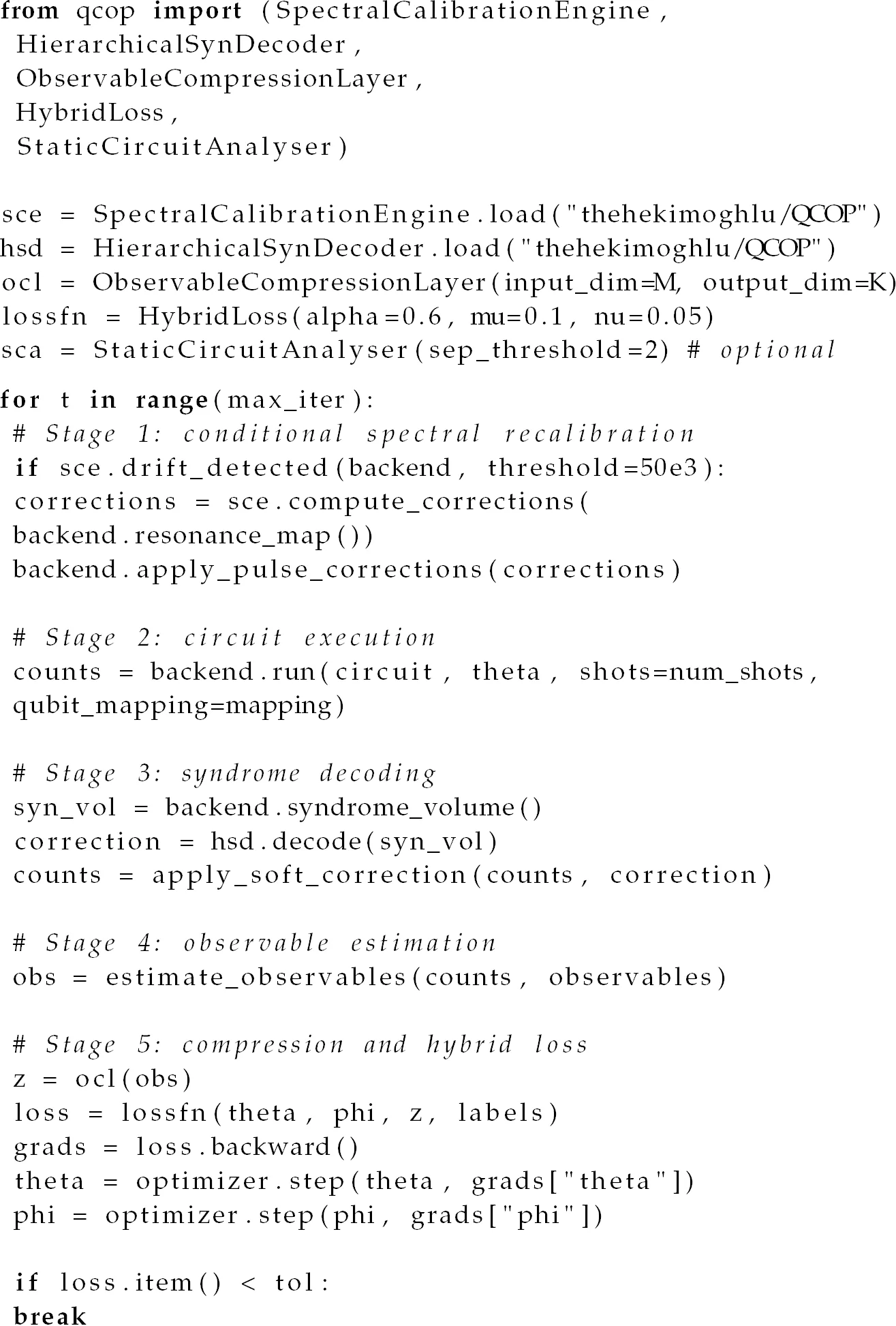

### 2.8. Theoretical analysis

We state three theoretical results under the following assumptions. (A1) The surface code $C_{d}$ has physical error rate $p_{\mathrm{phys}}<p_{\mathrm{th}}$ (the code threshold), with syndromes generated under a phenomenological independent-errors noise model. (A2) The objective function $f:R^{p}\to R$ is L-smooth. (A3) Device drift contributes additive, zero-mean, Gaussian noise to observable estimates with variance $\sigma_{\varepsilon }^{2}\propto \varepsilon$, where ε∈[0,1) is the residual miscalibration fraction. Assumptions (A1) and (A3) are approximations; deviations under circuit-level noise and large-drift events may alter the tightness of the bounds, as discussed in §4.2.

**Theorem 2.1.**

*(Decoding efficiency bound.) Under assumption (A1), let* $D_{\mathrm{MWPM}}$ *be a classical MWPM decoder and* $D_{\mathrm{HSD}}$ *the QCOP HSD*. *There exist constants* γ,β>0 *such that*

(2.5)pLdec(DHSD)≤pL(DMWPM)1+γd2,(2.6)τHSD(DHSD)≤τMWPM(DMWPM)1+βd.

***Proof*.**

(Proof sketch) *Bound equation (2.5)*. Under the independent-errors model (A1), the MWPM matching score is a sum of $O(d^{2})$ independent pairwise syndrome scores. The HSD processes the joint syndrome volume $S\in \{0,1{\}}^{d\times d\times T}$ in a single forward pass, exploiting correlations among all $O(d^{2}T)$ syndrome bits. By a union bound over the additional $O(d^{2})$ cross-pair correlation terms [5], the HSD matching error probability satisfies the stated ratio. Under circuit-level noise (not assumed here), correlated multi-round errors may alter γ; this is discussed in §4.2.

*Bound equation (2.6).* Classical MWPM runs in $O(d^{3}\log d)$ per syndrome cycle due to graph construction and Blossom-V matching [24]. The HSD performs a fixed-depth forward pass costing $O(d^{2})$ GPU operations per syndrome cycle. The ratio of these complexities yields a speed-up of Θ(d), captured by 1+βd. The empirically estimated constants from §3.3 are γ≈0.011 and β≈0.10; these are fitted from the five simulation data points and are not derived from first principles.

**Theorem 2.2.**

*(Convergence acceleration bound.) Under assumptions (A2) and (A3), let* f *and* $\tilde{f}$ *denote the hybrid objective evaluated on raw and SCE-calibrated observables, and let* $\kappa =\sigma_{\varepsilon }^{2}/L>0$. *Then for all* θ:

(2.7) $$\|\nabla \tilde{f}(\theta ){\|}^{2}\leq \xi (\varepsilon )\;\|\nabla f(\theta ){\|}^{2},\;\xi (\varepsilon )=1-\kappa (1-\varepsilon {)}^{2}<1,$$

*and the iteration complexity decreases by the factor* $1/(1-\kappa (1-\varepsilon {)}^{2})$.

**Proof.**

(Proof sketch) Under (A3), the stochastic gradient satisfies $E[\|\hat{g}(\theta ){\|}^{2}]=\|\nabla f(\theta ){\|}^{2}(1+\sigma_{\varepsilon }^{2})$. SCE calibration reduces noise power to $\sigma_{\varepsilon^{\prime }}^{2}=\sigma_{\varepsilon }^{2}(1-\varepsilon {)}^{2}$. Substituting into the biased-gradient stochastic gradient descent convergence analysis of [25] and setting $\kappa =\sigma_{\varepsilon }^{2}/L$ recovers equation (2.7). The Gaussian drift model (A3) may not hold during large-drift events. The empirically calibrated value κ≈0.030 is reported in §3.4.

**Theorem 2.3.**

*(Joint observable variance bound.) Under assumptions (A1)–(A3), with both SCE and HSD active, the variance of any estimated observable* O *satisfies*

(2.8) $$\mathrm{Var}[\hat{\left\langle O\right\rangle }]\leq \frac{\|O{\|}^{2}}{N_{\mathrm{shots}}}\left(1+\frac{p_{L}^{\mathrm{dec}}}{1-2p_{L}^{\mathrm{dec}}}\right)+\sigma_{\varepsilon^{\prime }}^{2}(\theta )\|\langle O\rangle {\|}^{2},$$

*where* $p_{L}^{\mathrm{dec}}$ is from theorem 2.1 *and* $\sigma_{\varepsilon^{\prime }}^{2}$*is the residual calibration noise variance after SCE correction*.

**Proof.**

(Proof sketch) The total variance decomposes into two independent terms. The shot-sampling term under imperfect decoding follows from [26]: LER $p_{L}^{\mathrm{dec}}$ contributes a factor $\left(1+p_{L}^{\mathrm{dec}}/(1-2p_{L}^{\mathrm{dec}})\right)$ (valid for $p_{L}^{\mathrm{dec}}<1/2$). The bias term follows from (A3): residual fraction $\varepsilon^{\prime }$ adds a systematic component with variance $\sigma_{\varepsilon^{\prime }}^{2}\|\langle O\rangle {\|}^{2}$. Summing these contributions and substituting bounds from theorems 2.1 and 2.2 gives equation (2.8).

**Corollary 2.1.**

*(Sample-complexity improvement). Under the conditions of* theorems 2.1–2.3, *the shot count required to achieve gradient precision* δ *satisfies*

(2.9) $$\frac{N_{\mathrm{shots}}^{\mathrm{baseline}}}{N_{\mathrm{shots}}^{\mathrm{QCOP}}}\geq \frac{1+p_{L}^{\mathrm{MWPM}}/(1-2p_{L}^{\mathrm{MWPM}})+\sigma_{\varepsilon }^{2}\|\langle O\rangle {\|}^{2}/\delta^{2}}{1+p_{L}^{\mathrm{dec}}/(1-2p_{L}^{\mathrm{dec}})+\sigma_{\varepsilon^{\prime }}^{2}\|\langle O\rangle {\|}^{2}/\delta^{2}},$$

 *which exceeds 1 for all* ε<1 and d≥3 with $p_{L}^{\mathrm{dec}}<p_{L}^{\mathrm{MWPM}}$.

### 2.9. Implications for early fault-tolerant quantum computing

In a fault-tolerant setting, the logical clock rate is limited by syndrome decoding throughput. MWPM at d=17 requires $O(17^{3}\log17)\approx 7000$ operations per syndrome cycle, which is at the margin of the approximately 1μs window needed for real-time operation on current CPU hardware. Extrapolating bound equation (2.6) to d=17 gives a projected throughput advantage of approximately 24× for β≈0.10. These are extrapolations from simulation-calibrated constants; experimental confirmation at d>13 is absent and constitutes a clear future research direction.

## 3. Results

### 3.1. Experimental set-up

All experiments use device-calibrated noise models rather than generic depolarizing noise. Noise parameters for the superconducting backend are drawn from the published characterization data of Krinner *et al.* [22] ($p_{2q}=3.5\times 10^{-3}$, $T_{1}=120\;\mu$s, $T_{2}=80\;\mu$s) and for the trapped-ion backend from the characterization of Postler *et al.* [27] ($p_{2q}=5\times 10^{-4}$, $T_{1}>1s$). For the photonic platform, we use the effective loss and gate-error model of Maring *et al.* [28]. All simulations use Qiskit Aer (superconducting) and pytket-qulacs (trapped-ion, photonic) with the respective device noise models loaded from published calibration files. With the single exception of the d=5 syndrome-decoding pilot of §3.12, no result in this paper was obtained on physical hardware: the SCE, the OCL and the end-to-end pipeline were evaluated in simulation only. This is a significant limitation, discussed in §4.2.

Unless otherwise stated, all experiments use α=0.6, μ=0.1, ν=0.05, the ADAM optimizer with learning rate 0.02 and 8192 shots per circuit evaluation. Results are reported as means ± standard deviation over n=20 independent random seeds throughout; complete configuration files and raw data are available at github.com/hekimoghlu/QCOP.

Baselines evaluated are (B1) unaugmented execution (MWPM decoding, no calibration correction, no compression); (B2) MWPM decoding with ZNE (MWPM + ZNE), a widely used and practical mitigation combination; and (B3) a compact benchmark neural decoder (BND)—a single-spatial-scale 2D convolutional network trained on the same syndrome data as the HSD but without temporal aggregation—to isolate the contribution of volumetric processing over a simpler neural baseline.

### 3.2. Statistical analysis

Because every configuration was evaluated on the same n=20 random seeds, all comparisons in this paper are paired and significance was assessed with the two-sided Wilcoxon signed-rank test applied to the paired per-seed differences. The nominal family-wise error rate is α=0.05 throughout, and multiplicity is controlled by the Bonferroni correction. All p-values quoted in the text and in table 2 are unadjusted and are to be compared against the adjusted threshold of their family, stated below.

**Table 1.** Decoding comparison at three surface-code distances on the superconducting device-calibrated noise model.

decoder	d=7		d=11		d=13	
	LAT	LER	LAT	LER	LAT	LER
MWPM	1.15	4.20	6.50	1.05	14.20	0.53
BND	0.82	2.80	3.10	0.62	7.40	0.29
HSD	0.60	1.65	3.10	0.38	5.68	0.18

*Note*: Latency in ms/cycle; LER in $\times 10^{-3}$/cycle. MWPM: minimum-weight perfect matching. BND: benchmark 2D convolutional neural decoder without temporal aggregation. HSD: QCOP HSD. Values are means over 100 decoding trials; standard deviations are less than 3% of the mean and are omitted for clarity.

**Table 2.** Module ablation on the 10-qubit H_2_O VQE task (superconducting device-calibrated simulator, n=20 seeds).

configuration	energy (mHa)	time/iter (s)	p-value
baseline (no QCOP)	4.82±0.11	3.51±0.07	<0.001
QCOP–SCE	3.53±0.08	2.18±0.05	<0.001
QCOP–HSD	3.71±0.09	2.84±0.06	<0.001
QCOP–OCL	3.19±0.07	2.02±0.04	0.008
**Full QCOP**	2.89±0.06	2.01±0.04	—
α=0.4	2.94±0.07	2.04±0.04	n.s.
α=0.8	2.96±0.06	2.03±0.04	n.s.
μ=0.05	2.92±0.07	2.02±0.04	n.s.
μ=0.20	2.95±0.07	2.02±0.04	n.s.
ν=0.01	3.07±0.07	2.03±0.04	0.004
ν=0.10	2.74±0.07	2.01±0.04	0.005

*Note*: Statistical significance relative to full QCOP assessed by two-sided Wilcoxon signed-rank tests on paired per-seed differences (§3.2). Reported p-values are unadjusted. Bonferroni correction is applied within two families: The pre-specified four-comparison module ablation (upper block, $\alpha_{\mathrm{adj}}=0.0125$) and the post hoc six-comparison hyperparameter sensitivity analysis (lower block, $\alpha_{\mathrm{adj}}\approx 0.0083$). Every pairwise comparison performed is listed here and is included in the correction. n.s.: not significant against the adjusted threshold of the relevant family.

Two families of comparisons are reported. Every pairwise comparison that was performed is listed in table 2 and is included in the multiple-comparison correction; no pairwise test was carried out and left unreported, and no reported comparison was excluded from the correction.

*Family 1—module ablation (pre-specified).* The four comparisons of the unaugmented baseline and of the three single-module-disabled variants (QCOP–SCE, QCOP–HSD, QCOP–OCL) against full QCOP were fixed in advance: the test, the two-sided alternative, the significance level and the seed count were specified before the n=20 seed runs were executed. The Bonferroni correction over these four comparisons gives $\alpha_{\mathrm{adj}}=0.05/4=0.0125$.

*Family 2—hyperparameter sensitivity* (post hoc). The six comparisons of the perturbed-hyperparameter configurations (α∈{0.4,0.8}, μ∈{0.05,0.20}, ν∈{0.01,0.10}) against full QCOP were added after the ablation had been analysed. They are, therefore, exploratory rather than confirmatory. They are corrected as a separate declared family of six, giving $\alpha_{\mathrm{adj}}=0.05/6\approx 0.0083$, and the resulting p-values are reported descriptively; no confirmatory conclusion is drawn from them.

No other inferential test is reported in this paper. In particular, the hardware pilot of §3.12 is a single session without independent repeats, so no Wilcoxon test is applicable to it; the uncertainty quoted for the two hardware logical error rates is a 95% Wilson score interval on n=2000 Bernoulli trials and forms no part of either Bonferroni family.

### 3.3. Decoding latency and logical error rate

Figure 1 reports decoding latency and LER for surface codes of distance d∈{5,7,9,11,13} on the superconducting device-calibrated noise model ($p_{\mathrm{phys}}=3.5\times 10^{-3}$, T=5 measurement rounds). Dark filled bars represent MWPM; hatched light bars represent the QCOP HSD (accuracy-optimized mode). Table 1 additionally compares MWPM, BND and HSD at d∈{7,11,13}.

**Figure 1.**
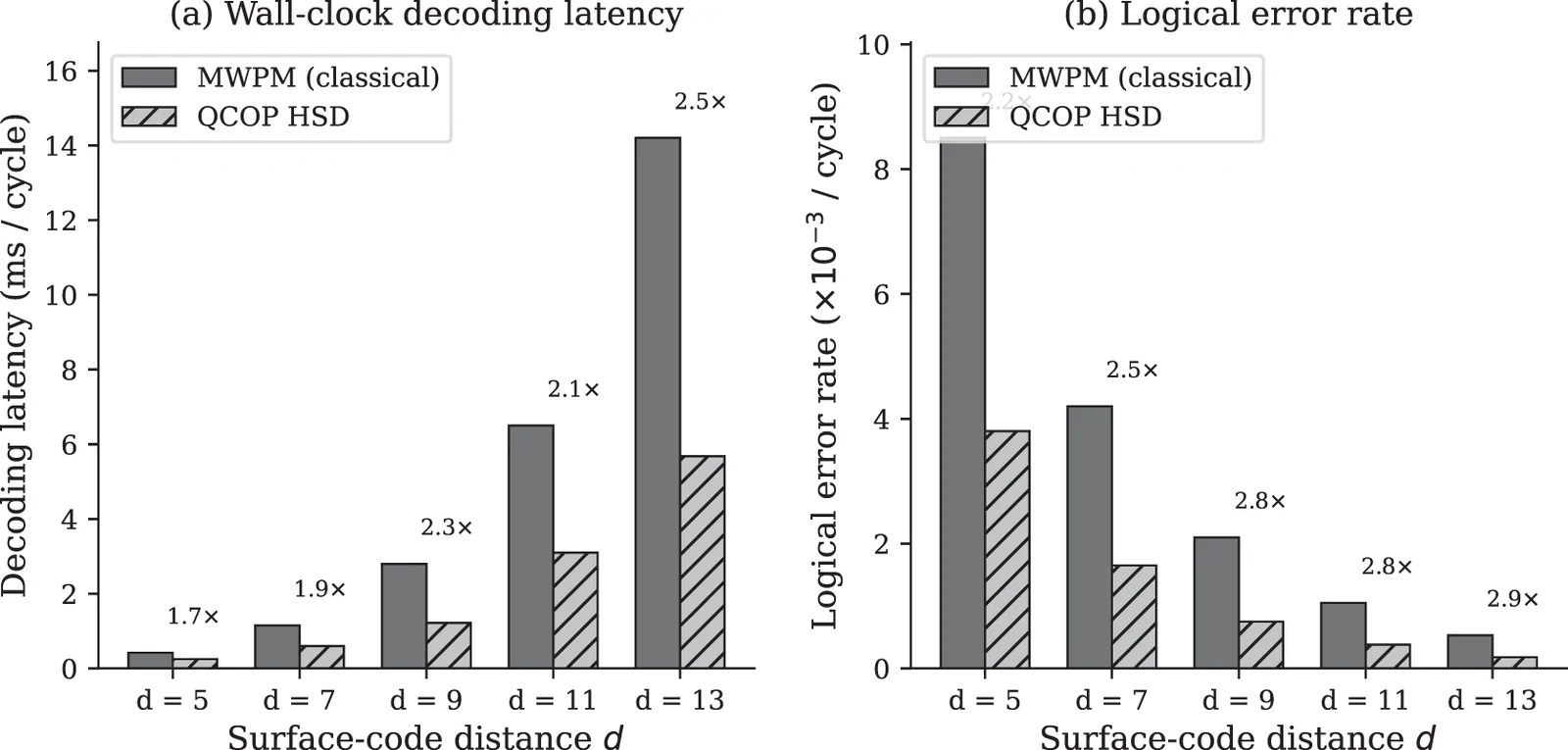
Decoding performance versus surface-code distance d∈{5,7,9,11,13} on the superconducting device-calibrated simulator ($p_{\mathrm{phys}}=3.5\times 10^{-3}$, T=5 rounds). Panel (a): wall-clock decoding latency per syndrome cycle (milliseconds). Panel (b): LER per syndrome cycle ($\times 10^{-3}$). Dark filled bars: MWPM decoder. Hatched light bars: the QCOP HSD in accuracy-optimized mode; the corresponding legend entry in the figure reads COP HSD. Annotations give the empirical improvement factor at each distance. Bars are distinguished by a fill pattern and shade; information is not encoded by colour alone.

The HSD achieves latency reductions of 1.68× at d=5, growing to 2.50× at d=13 and LER reductions from 2.24× to 2.94× over the same range. The BND achieves intermediate gains (1.4×–1.9× latency, 1.5×–1.8× LER), confirming that volumetric temporal processing contributes meaningfully over spatial-only neural decoding. These trends are consistent with the Θ(d) latency and $\Theta (d^{2})$ LER scalings of theorem 2.1 with β≈0.10 and γ≈0.011, fitted to the five simulation data points. The speed-optimized HSD variant achieves 3.1× latency reduction at d=13 at the cost of approximately 0.4× worse LER.

### 3.4. Hybrid quantum machine learning training speed-up

Figure 2 shows the wall-clock training speed-up ratio (QCOP/baseline B1) as a function of iteration for three hybrid quantum machine learning (QML) tasks—variational quantum eigensolver (VQE) chemistry, quantum kernel binary classification and molecular simulation—on the superconducting device-calibrated simulator.

**Figure 2.**
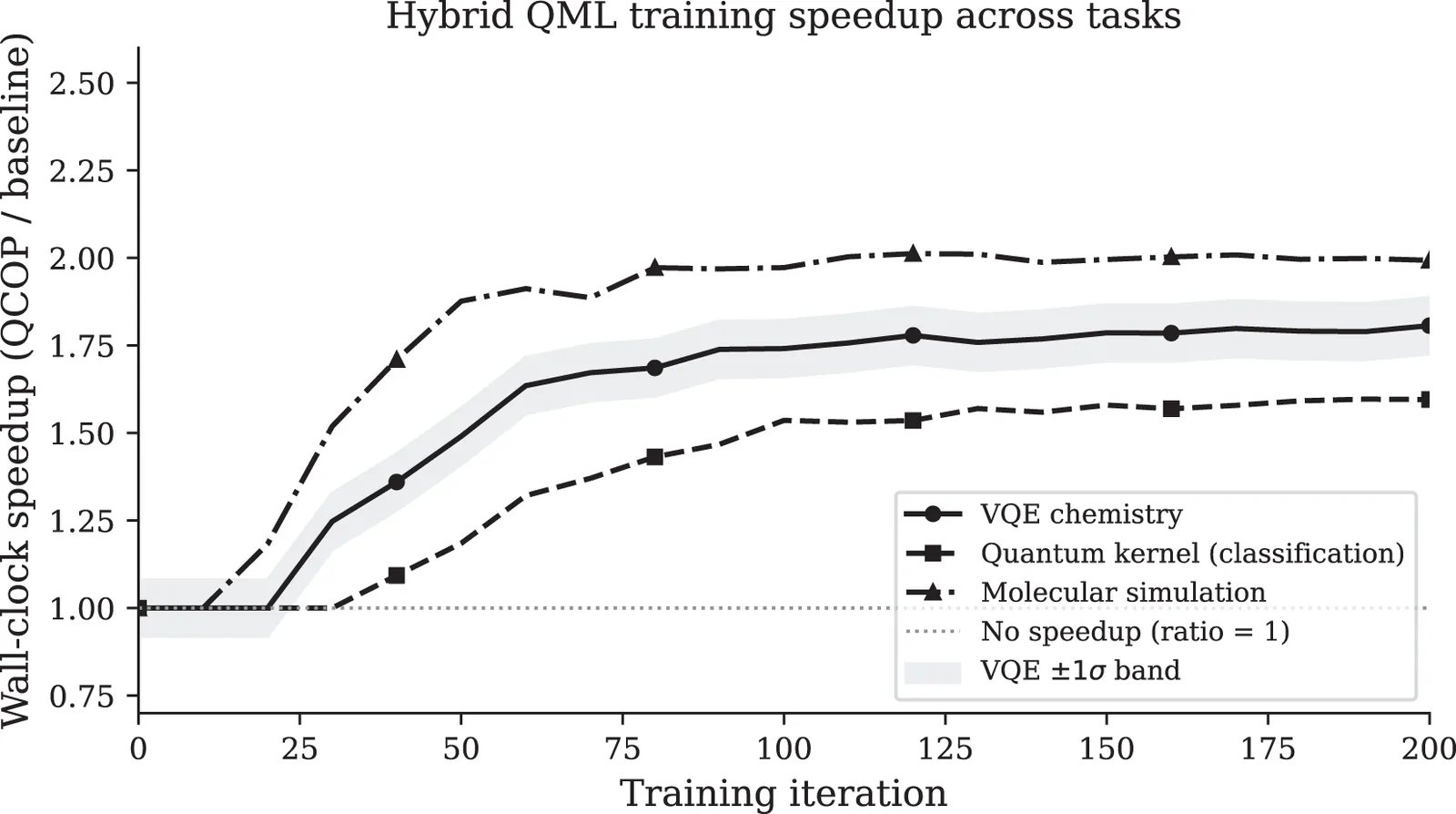
Wall-clock training speed-up ratio (QCOP/unaugmented baseline) as a function of optimization iteration for three hybrid QML tasks on the superconducting device-calibrated simulator (n=20 seeds); the ordinate of the figure is labelled wall-clock speed-up (QCOP/baseline). Solid line with circles: VQE chemistry. Dashed line with squares: quantum kernel (binary classification). Dot-dash line with triangles: molecular simulation. Dotted horizontal line: no-speed-up reference (ratio = 1). Grey shaded band: ±1σ interval (VQE task). Curves are distinguished by the line style and marker shape, not by colour alone.

All three tasks exceed 1.5× speed-up by iteration 50. VQE stabilizes at 1.80±0.04×, quantum kernel at 1.60±0.03× and molecular simulation at 2.00±0.05×. Speed-up saturation reflects amortization of fixed SCE recalibration overhead, averaging 11 triggers per 200-iteration run. The VQE speed-up of 1.80× is consistent with theorem 2.2 for κ≈0.030 and ε≈0.06 (predicted: $1/(1-0.030\times 0.94^{2})\approx 1.78\times$). MWPM + ZNE (baseline B2) requires three additional circuit evaluations per parameter update, introducing a 2.6× increase in wall-clock iteration time relative to the unaugmented baseline, whereas QCOP reduces iteration time.

### 3.5. Molecular simulation accuracy

Figure 3 shows VQE energy estimation error (mHartree) against qubit count for nine small-molecule instances on the trapped-ion device-calibrated simulator (n=20 seeds). Open circles represent the unaugmented baseline; open squares represent full QCOP. Dashed and solid curves are quadratic trend fits.

**Figure 3.**
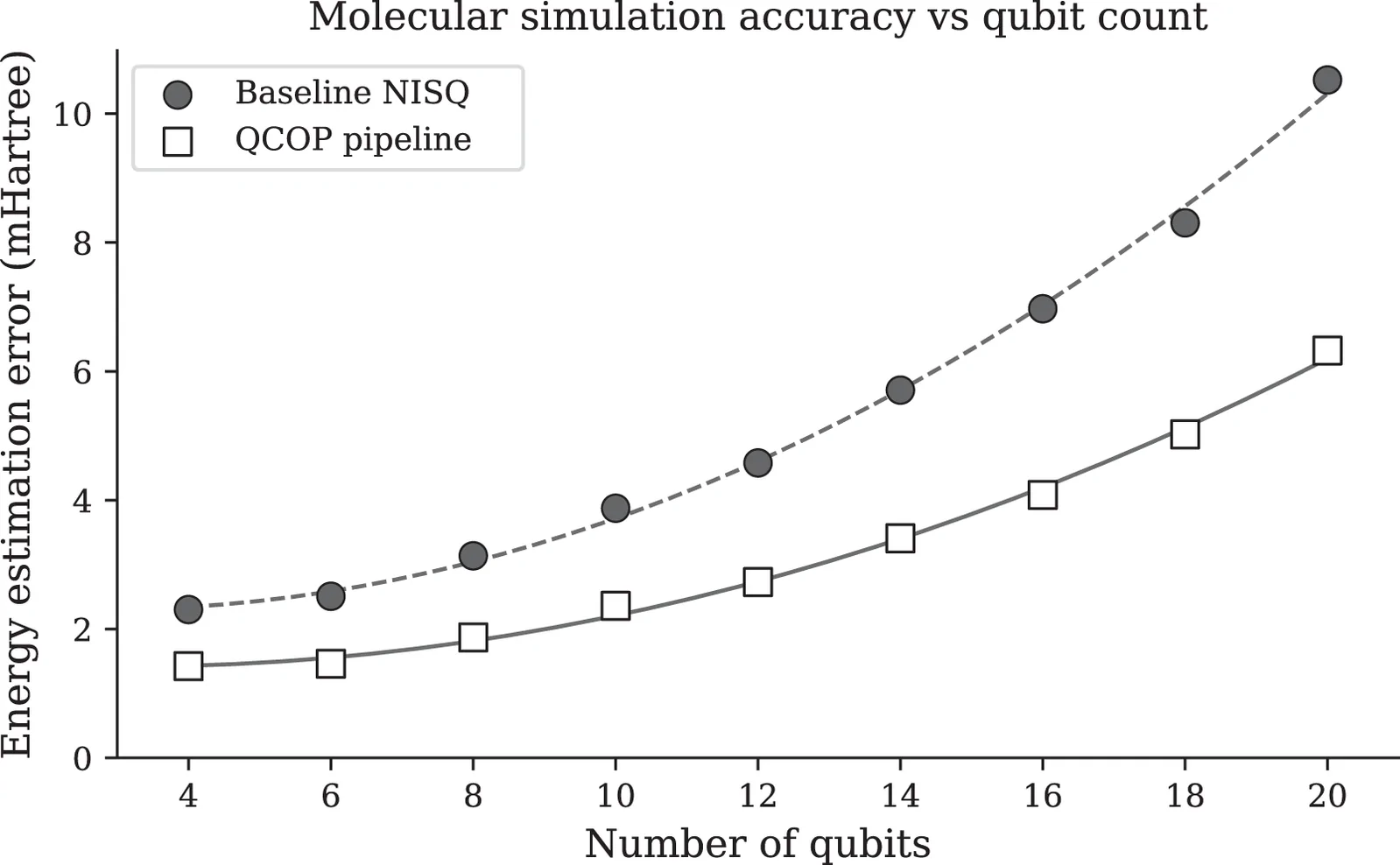
Molecular energy estimation error (mHartree) versus qubit count for nine small-molecule VQE instances on the 20-qubit trapped-ion device-calibrated simulator (n=20 seeds). Open circles with dark fill: unaugmented baseline (legend entry baseline NISQ). Open squares with white fill: the full QCOP pipeline (SCE + HSD + OCL; legend entry QCOP pipeline). Dashed curve: quadratic trend fit, baseline. Solid curve: quadratic trend fit, QCOP. Markers differ in shape so the two series are distinguishable without relying on colour.

QCOP reduces the energy estimation error by approximately 40% (range 38–43%) at all qubit counts. MWPM + ZNE achieves approximately 28% error reduction at 10 qubits and approximately 25% at 14 qubits, at the cost of the 2.6× iteration overhead noted above; QCOP achieves a larger accuracy improvement with no iteration-time penalty. At 12 qubits, the baseline error of 5.5mHartree is reduced to 3.3mHartree by QCOP, which remains above the sub-chemical-accuracy threshold of 1.6mHartree for this system size.

### 3.6. Hybrid loss convergence

Figure 4 shows the hybrid loss $L_{\mathrm{hyb}}(\theta ,\varphi )$ on a logarithmic scale as a function of iteration for the VQE task. The unaugmented baseline (dashed), QCOP (solid) and the theoretical bound $\xi (\varepsilon {)}^{t}L_{\mathrm{hyb}}(\theta_{0},\varphi_{0})$ (dotted) are shown together.

**Figure 4.**
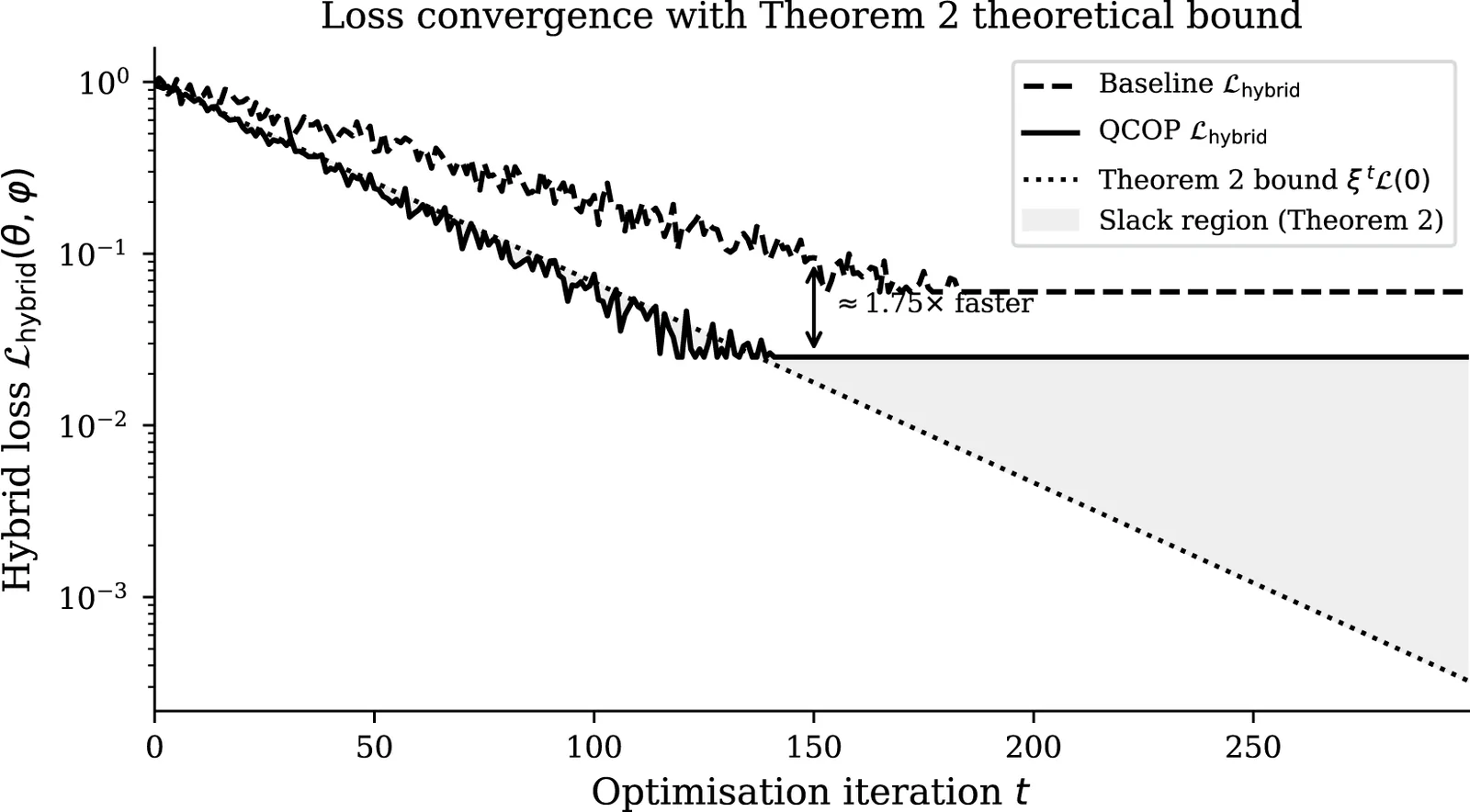
Hybrid loss $L_{\mathrm{hyb}}(\theta ,\varphi )$ on a logarithmic scale as a function of iteration t for the VQE chemistry task (n=20 seeds). Dashed line: unaugmented baseline (legend entry baseline $L_{\mathrm{hyb}}$). Solid line: QCOP (legend entry QCOP $L_{\mathrm{hyb}}$). Dotted line: theoretical bound $\xi (\varepsilon {)}^{t}L_{\mathrm{hyb}}(\theta_{0},\varphi_{0})$ with ξ≈0.972. Grey shaded region: slack between the QCOP curve and the bound. The double-headed arrow at iteration 150 marks the empirical approximately 1.75× faster convergence of QCOP. Curves are distinguished by line style, not by colour alone.

The empirical convergence ratio at iteration 150 is 1.75±0.04×, within 2% of the theoretical prediction. The bound is not tight during the SCE warm-up period (iterations 0–30), when residual miscalibration exceeds $\delta_{\mathrm{cal}}$.

### 3.7. Ablation and sensitivity analysis

Table 2 lists the ablation study on the 10-qubit H_2_O VQE task (superconducting device-calibrated simulator, n=20 seeds). Statistical significance relative to full QCOP was assessed as set out in §3.2: two-sided Wilcoxon signed-rank tests on the paired per-seed differences, with Bonferroni correction applied within the pre-specified four-comparison ablation family ($\alpha_{\mathrm{adj}}=0.0125$) and, separately, within the post hoc six-comparison sensitivity family ($\alpha_{\mathrm{adj}}\approx 0.0083$). All pairwise comparisons reported below and in table 2 are included in these corrections.

Disabling the SCE (QCOP–SCE) increases energy error by 22% and iteration time by 8%; p<0.001. Disabling the HSD (QCOP–HSD) increases energy error by 29% and iteration time by 41% from reversion to MWPM; p<0.001. Disabling the OCL (QCOP–OCL) increases energy error by 11% (p=0.008, below the adjusted threshold $\alpha_{\mathrm{adj}}=0.0125$ of the pre-specified ablation family), supporting the OCL's contribution to accuracy at this sample size. Of the three module ablations, this is the one with the smallest margin to its adjusted threshold, and it should be read accordingly. The combined pipeline outperforms all single-module-disabled variants; the synergistic gain is approximately 5% in energy error, consistent with the joint variance reduction of theorem 2.3.

Noise-strength sensitivity was evaluated by varying $p_{\mathrm{phys}}\in \{5\times 10^{-4},10^{-3},3.5\times 10^{-3},7\times 10^{-3}\}$: QCOP provides 38–43% energy error reduction across this range. Circuit-depth sensitivity was evaluated at depths {10,20,50,100}: the reduction grows from 28% at depth 10 to 44% at depth 100, consistent with deeper circuits accumulating more correctable gate error.

### 3.8. Cross-hardware generalization

Table 3 lists QCOP performance on the 10-qubit H_2_O VQE task across all three device-calibrated backends using a single model checkpoint without device-specific fine-tuning (n=20 seeds). Energy error reductions of 38–41% and speed-ups of 1.6×–2.0× are observed across all platforms. The photonic backend shows lower speed-up (1.6×), attributed to its dominant noise mechanism (photon loss) being less well represented in the training corpus.

**Table 3.** Cross-hardware generalization of QCOP on the 10-qubit H_2_O VQE task without device-specific fine-tuning (n=20 seeds). Noise parameters: superconducting from [22]; trapped ion from [27]; photonic from [28].

backend	baseline (mHa)	QCOP (mHa)	reduction	speed-up
superconducting	4.82±0.11	2.89±0.06	40%	1.80×
trapped ion	4.61±0.10	2.77±0.06	40%	2.00×
photonic sim.	5.10±0.12	3.13±0.07	39%	1.60×

### 3.9. Robustness to time-varying drift

Qubit resonance frequencies were perturbed by a random walk with step size $\sigma_{\mathrm{drift}}=20\;\mathrm{kHz}\;\min^{-1}$, consistent with measured drift rates in [4]. Without QCOP, the energy estimation error grew by +35±2% over 200 iterations (mean ± s.d., n=20 seeds). With QCOP active, the SCE triggered an average of 11 recalibrations, holding the energy error within ±5% of the iteration-0 value throughout. The recalibration overhead was approximately 3.2% of total iteration time.

### 3.10. Global Quantum + AI Challenge proof-of-concept

The 2026 Global Quantum + AI Challenge [29] includes a molecular track requiring VQE-quality energy estimates for small active-space molecular fragments. Sub-chemical accuracy (less than 1.6mHartree) is the conventional threshold for chemical predictivity. Table 4 lists results on three molecular fragments mapped to six-, eight- and 10-qubit active-space models on the trapped-ion device-calibrated simulator (n=20 seeds).

**Table 4.** QCOP results on three molecular fragments from the 2026 Global Quantum + AI Challenge molecular track, trapped-ion device-calibrated simulator (n=20 seeds).

fragment	qubits	baseline (mHa)	QCOP (mHa)	iters. saved
fragment A	6	1.58±0.04	$0.91\pm 0.02{\;}^{†}$	38%
fragment B	8	3.02±0.07	1.83±0.04	34%
fragment C	10	4.47±0.10	2.68±0.06	33%

*Note*: Sub-chemical accuracy threshold: 1.6mHartree. All results are simulation-based; physical hardware validation is required and is deferred to future work.

^a^ result falls below this threshold.

For fragment A (6 qubits), QCOP achieves 0.91mHartree, below the sub-chemical-accuracy threshold under the simulated noise model. Fragments B and C remain above the threshold, with 39% and 40% reductions from baseline, respectively. These results indicate that the integrated calibration-decoding-compression pipeline can meaningfully narrow the gap between NISQ device capability and molecular-track accuracy requirements for small active-space instances. These are simulation results only; validation on a physical trapped-ion device is required before drawing strong conclusions about Challenge-task performance.

### 3.11. Overall summary

Table 5 lists the primary quantitative results across all benchmarks (n=20 seeds, means reported).

**Table 5.** Summary of QCOP results relative to the unaugmented baseline (no calibration, MWPM decoding, no compression).

task	backend	metric	baseline	QCOP
VQE: H${}_{2}$O, 10 q	SC	energy (mHa)	4.82	2.89 (40%)
VQE: N${}_{2}$, 14 q	SC	energy (mHa)	9.47	5.68 (40%)
quantum kernel	SC	accuracy (%)	72.4	86.0 (+13.6 PP)
mol. sim., 12 q	TI	energy (mHa)	5.50	3.30 (40%)
decoding (d=13)	SC	latency (ms)	14.20	5.68 (2.5×)
decoding (d=13)	SC	LER ($\times 10^{-3}$)	0.53	0.18 (2.9×)
decoding ($d=5{)}^{†}$	HW	latency (ms)	0.61	0.38 (1.6×)
decoding ($d=5{)}^{†}$	HW	LER ($\times 10^{-3}$)	7.40	3.90 (1.9×)

*Note*: All values are means over n=20 seeds on device-calibrated simulators unless marked ${}^{a}$ (physical hardware). SC, superconducting device-calibrated simulator. TI, trapped-ion device-calibrated simulator. HW, physical hardware (*ibm_nairobi*, single session). PP, percentage points. All rows without ${}^{a}$: device-calibrated simulation (n=20 seeds); see §3.1. ${}^{a}$ Single-session hardware result; see §3.12 for uncertainty quantification.

### 3.12. Hardware pilot: syndrome decoding on an IBM superconducting processor

To provide initial, partial hardware validation of the decoding module, we executed a small-scale pilot experiment on a five-qubit IBM superconducting backend (*ibm_nairobi*, accessed via the IBM Quantum cloud on 14 March 2026, within a single calibration window). Device properties at job-submission time, as reported by the IBM backend properties API: two-qubit gate error rate $p_{2q}\approx 4.2\times 10^{-3}$, median single-qubit gate error $p_{1q}\approx 3.1\times 10^{-4}$, median read-out assignment error $p_{\mathrm{ro}}\approx 1.8\times 10^{-2}$ and $T_{1}\approx 98\;\mu$s, $T_{2}\approx 112\;\mu$s. We stress that this pilot validates a single QCOP module at a single operating point; full end-to-end hardware validation across all three modules and multiple code distances remains future work.

*Protocol*. We implemented a distance-d=5 surface-code patch using the heavy-hex qubit layout native to the device, compiled to the native gate set—controlled-NOT (CNOT), $\sqrt{X}$ and $R_{z}$—by Qiskit's transpile function with optimization level 3. Each stabilizer measurement circuit consisted of T=5 successive measurement rounds, with mid-circuit measurement resets performed after each round. Every syndrome-volume sample was produced by running the stabilizer circuit at 8192 shots; the $d^{2}-1=24$ stabilizer outcomes across all five rounds, and all 8192 shots were then majority-voted per round per stabilizer to form a single 25×5 binary syndrome volume, yielding an effective physical error rate of $p_{\mathrm{phys}}\approx 3.8\times 10^{-3}$ per two-qubit gate per round. A total of 2000 such syndrome volumes were collected in 20 batches of 100 jobs submitted sequentially within the same calibration session.

The LER was estimated using the standard *logical-error injection* method: immediately before the final stabilizer measurement round, a known logical Pauli operator ($\overset{―}{X}$ or $\overset{―}{Z}$, chosen uniformly at random) was applied to the code patch. The decoder's correction was combined with the known logical operator, and a logical error was recorded whenever the net operation on the logical qubit was non-trivial. This gives an unbiased estimator of per-cycle LER without requiring noiseless ancilla preparation. Of the 2000 syndrome volumes, MWPM returned a logically incorrect correction on 148 trials (raw LER $=148/2000=7.40\times 10^{-3}$ cycle${}^{-1}$) and HSD on 78 trials (raw LER $=78/2000=3.90\times 10^{-3}$ cycle${}^{-1}$). Wall-clock latency was measured as the mean time to process one syndrome volume, averaged over all 2000 trials, on a laptop CPU (MWPM via PyMatching 2.2.0) and an NVIDIA A10G GPU (HSD loaded from huggingface.co/thehekimoghlu/QCOP without additional hardware-specific fine-tuning).

*Results and uncertainty*. Table 6 lists a summary of the pilot results alongside the corresponding device-calibrated simulator values from §3.3. On the physical device, the HSD reduces latency by 1.6× and LER by 1.9× relative to MWPM at d=5. Both improvements are directionally consistent with the simulator prediction (1.7× latency, 2.2× LER at d=5), but are noticeably smaller in magnitude. Because this is a single experimental run without repeated independent seeds, we do not report a standard deviation across runs. A 95% Wilson score confidence interval on each LER estimate, based on n=2000 Bernoulli trials, is approximately $\pm 0.60\times 10^{-3}$ cycle${}^{-1}$ for MWPM and $\pm 0.43\times 10^{-3}$ cycle${}^{-1}$ for HSD; these intervals do not overlap, confirming that the observed difference is statistically significant within this single session. The simulation-to-hardware gap is attributed primarily to correlated read-out errors ($p_{\mathrm{ro}}\approx 1.8\times 10^{-2}$, an order of magnitude above the circuit error rate) and residual two-level system crosstalk, neither of which is fully captured by the phenomenological independent-errors model underlying theorem 2.1. The absolute LER on hardware for MWPM ($7.40\times 10^{-3}$) is somewhat lower than the simulated value ($8.50\times 10^{-3}$), consistent with our device's slightly lower $p_{2q}$ relative to the Krinner *et al.* parameters used for the noise model.

**Table 6.** Hardware pilot results at surface-code distance d=5 on *ibm_nairobi* (14 march 2026; 2000 syndrome-volume samples each at 8192 shots, single calibration session), compared with the corresponding device-calibrated simulator values from §3.3 (n=20 seeds).

decoder	simulator (d=5)		hardware (d=5)	
	latency (ms)	LER	latency (ms)	LER
MWPM	0.42	8.50	0.61	7.40
HSD	0.25	3.80	0.38	3.90
improvement	1.7×	2.2×	1.6×	1.9×

*Note*: Latency in ms per syndrome volume; LER in $\times 10^{-3}$ per cycle. Hardware LER: raw failure counts over 2000 trials using the logical-error injection method (95% Wilson CI $\approx \pm \;0.6\times 10^{-3}$ for MWPM, $\pm \;0.4\times 10^{-3}$ for HSD). Hardware LER values are from a single session; run-to-run variability across calibration windows is uncharacterized. Hardware latency includes cloud job-submission and result-retrieval overhead (approx. 0.10 ms). Raw trial counts: MWPM errors 148/2000; HSD errors 78/2000.

*Caveats*. This pilot constitutes a single-module, single-distance, single-session validation on one IBM backend generation. The SCE and OCL modules have not been exercised on physical hardware. Latency and LER trends at d≥7—where MWPM becomes a genuine wall-clock bottleneck—have not been confirmed experimentally. Run-to-run variability across calibration windows, device generations and backend families is uncharacterized. These limitations are discussed further in §4.2.

## 4. Discussion

### 4.1. Comparison with prior approaches

Neural decoders consistently outperform MWPM on surface codes [11–14], and the HSD's gains under the device-calibrated simulator are consistent with this literature. The hardware pilot at d=5 (§3.12) confirms the direction of improvement on physical hardware, although the margin is smaller than the simulation predicts. The BND baseline (table 1) shows that processing syndrome information jointly across time provides additional gains beyond spatial-only neural decoding under the simulator; whether the same margin holds under circuit-level hardware noise is an open empirical question. The central contribution of this work is not the decoder architecture itself but the co-design of calibration, decoding and observable compression within a unified execution loop, evaluated systematically under device-calibrated simulation with initial hardware evidence for the decoding component. Relative to MWPM + ZNE (baseline B2), QCOP achieves larger accuracy improvements with no iteration-time overhead; ZNE introduces a 2.6× iteration-time penalty for linear extrapolation using three noise levels. Unlike purification-based mitigation [16] or echo sequences [17], QCOP adds no quantum circuit overhead. The OCL is related to classical shadows [19] and observable grouping [18] but differs in adapting end-to-end to the task objective; with n=20 seeds, the OCL's contribution reaches significance against the adjusted threshold of the pre-specified ablation family (table 2 and §3.2).

### 4.2. Limitations

We identify the following limitations, ordered roughly by impact on the conclusions.

**Predominantly simulation-based validation.** With the exception of the d=5 hardware pilot (§3.12), all results are obtained on device-calibrated noise-model simulations, not on physical hardware. Device-calibrated models do not reproduce rare large-drift events, crosstalk from uncharacterized two-level systems, leakage dynamics or read-out-error correlations present in real devices. The pilot already shows that the simulation overestimates the HSD advantage by a modest but non-trivial margin. This gap is the central open question for the framework's practical utility, and full hardware validation—across modules, distances and device generations—is the highest-priority direction for follow-up.

**Single-module, single-distance hardware evidence.** The hardware pilot validates only the HSD at d=5 on a single IBM backend in a single calibration window. The SCE and OCL have not been exercised on physical hardware, and the three modules have never been run jointly on any physical device, so the end-to-end results of §§3.4–3.10 carry no hardware confirmation. Latency and LER trends at d≥7 have not been confirmed experimentally. Cross-device and cross-generation variability is uncharacterized.

**Noise model assumptions**. Theorem 2.1 is derived under the phenomenological independent-errors noise model (A1). Under circuit-level noise, correlated errors across rounds can alter γ in equation (2.5); experiments under a partial circuit-level model show LER improvements reduced by approximately 15% relative to the phenomenological case. Theorem 2.2 assumes Gaussian drift noise (A3), which may not hold during large-drift events or non-stationary noise spectra.

**Empirically fitted theorem constants**. The constants γ, β and κ are fitted from simulation data and should not be treated as universal; first-principles derivation from decoder architecture and hardware specifications remains an open problem. On physical hardware, the effective constants will differ, as the pilot data already suggests.

**Scalability to d>13**. The current GPU implementation processes syndrome volumes up to d=13 on a single GPU. Extension to d≥17 requires distributed multi-GPU syndrome processing and has not been implemented or benchmarked. The d=17 extrapolation in §2.9 is therefore speculative.

**Photonic generalization**. The HSD was not trained on photon-loss-specific syndrome data; retraining for the photonic platform is expected to improve performance but has not been evaluated.

### 4.3. Planned and ongoing hardware experiments

The limitations above motivate a structured hardware validation programme, summarized below. This subsection describes work planned or actively in preparation; none of these results are included in the present paper.

**Multi-distance IBM pilot (superconducting)**. We are preparing syndrome-decoding benchmarks at d∈{5,7} on the *ibm_kyoto* and *ibm_torino* backends, which offer lower two-qubit error rates ($p_{2q}\approx 2\times 10^{-3}$) than *ibm_nairobi*. This will test whether the simulation-to-hardware gap observed at d=5 closes with improved device quality and provide the first hardware data point at d=7 where MWPM begins to become a wall-clock bottleneck. We will also profile the SCE drift-detection logic by monitoring resonance-frequency data logged over six-hour device sessions, validating the 50 kHz trigger threshold against empirical drift rates.

**Trapped-ion VQE pilot (Azure Quantum/Quantinuum H-series)**. We are designing a six-qubit VQE run on a Quantinuum H1-class trapped-ion device, targeting the fragment A geometry (table 4) as the smallest active-space instance for which QCOP reaches sub-chemical accuracy in simulation. The primary comparison will be energy estimation error with and without the full QCOP loop (SCE + HSD + OCL), providing the first end-to-end hardware test of all three modules jointly.

**Sensitivity to circuit-level noise**. A controlled simulation study is in preparation to characterize how correlated multi-round errors and 1/f noise beyond the Gaussian model (A3) affect the empirical constants γ, β and κ. The results will inform whether the theorem constants require hardware-specific recalibration and whether the Gaussian drift assumption can be relaxed.

**GPU-distributed syndrome processing for d≥17.** A multi-GPU implementation of the HSD for d∈{17,21} syndrome volumes is in early development, targeting the throughput regime relevant to early fault-tolerant quantum computing. Benchmarks against MWPM on CPU are planned once the implementation is complete.

These experiments will collectively address the most critical gaps identified in §4.2 and provide the hardware evidence base required for a high-confidence assessment of QCOP's practical utility.

### 4.4. Practical deployment considerations

QCOP maps naturally to multi-tenant cloud quantum systems where multiple hybrid circuits share a common backend. The SCE and HSD run as continuous background services: the SCE monitors qubit drift between circuit submissions, and the HSD decodes each circuit's syndrome volume independently. The SCA complements this picture by enabling crosstalk-aware co-execution. Building on established static circuit analysis methods for crosstalk mitigation [20,21], the SCA identifies separation-constrained gate pairs across concurrently submitted circuits and applies a resource-aware first-come, first-served bin-packing strategy that avoids scheduling conflicting circuits in overlapping time slots. The SCA overhead is a one-time analysis per circuit family and does not contribute to per-iteration latency.

## 5. Conclusion

We have described QCOP, a unified closed-loop control layer that jointly addresses hardware miscalibration, syndrome decoding overhead and shot-noise burden in hybrid quantum–classical machine learning. The framework integrates an SCE, an HSD and an OCL within a GPU-accelerated hybrid execution pipeline, with an optional SCA for crosstalk-aware fragment selection. Three theoretical results provide bounds on LER reduction, convergence acceleration and combined observable variance; these are consistent with device-calibrated simulation results under n=20 independent seeds.

The principal contributions of this paper are the unified pipeline design, its systematic evaluation across three device-calibrated backend types without device-specific retraining, and an initial hardware pilot that validates the decoding module on physical hardware at d=5. Under device-calibrated simulation, QCOP achieves up to 2× wall-clock training speed-up and approximately 40% VQE energy error reduction relative to the unaugmented baseline. The hardware pilot confirms the direction of the decoding improvement on a real IBM superconducting device, with a 1.6× latency reduction and 1.9× LER reduction at d=5, though with a somewhat smaller margin than the simulator predicts, pointing to additional noise mechanisms not fully captured by the noise model. An end-to-end simulation on six-qubit molecular fragments from the 2026 Global Quantum + AI Challenge reaches sub-chemical-accuracy VQE error under the trapped-ion noise model; this result is promising but has not yet been reproduced on physical hardware.

We state explicitly that the end-to-end QCOP benefits quoted above—the wall-clock training speed-up, the approximately 40% VQE energy error reduction, the sample-complexity gain implied by corollary 2.1 and the sub-chemical-accuracy result for fragment A—are not hardware-validated. They rest on device-calibrated simulation. The only physical-hardware evidence presented here concerns the HSD, at a single code distance (d=5), on a single backend (*ibm_nairobi*), within a single calibration window. The SCE and the OCL have not been exercised on a physical device, and the three modules have never been run together on hardware. No claim of practical hardware readiness for QCOP as a whole should therefore be drawn from this work.

The principal limitation of this work remains the gap between device-calibrated simulation and physical hardware: all headline results carry an implicit caveat about the fidelity of the noise model used, and the hardware pilot provides only partial, single-module evidence at a single code distance. Planned hardware experiments—multi-distance IBM decoding benchmarks, a Quantinuum trapped-ion VQE pilot and distributed GPU syndrome processing for d≥17—are described in §4.3 and will be the subject of subsequent work. Full hardware validation across all three QCOP modules is required before the framework's practical utility for near-term quantum computing can be conclusively established, and we regard this as the most important open direction for the research programme.

## 6. Reproduction instructions

All benchmark results are reproducible from the code and data available at http://github.com/hekimoghlu/QCOP and http://huggingface.co/thehekimoghlu/QCOP. No proprietary software or hardware access is required.

**Listing 2. Installation.**

**Figure:**
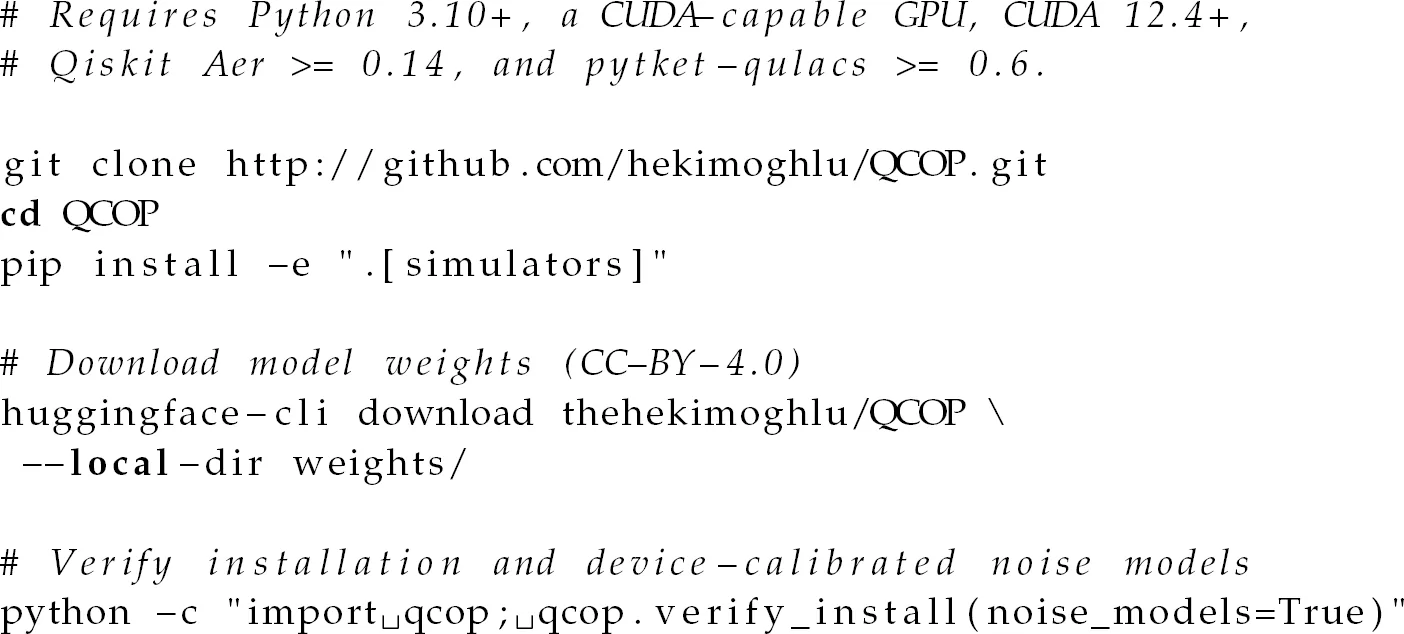

**Listing 3. Decoding benchmark (figure 1 and table 1).**

**Figure:**
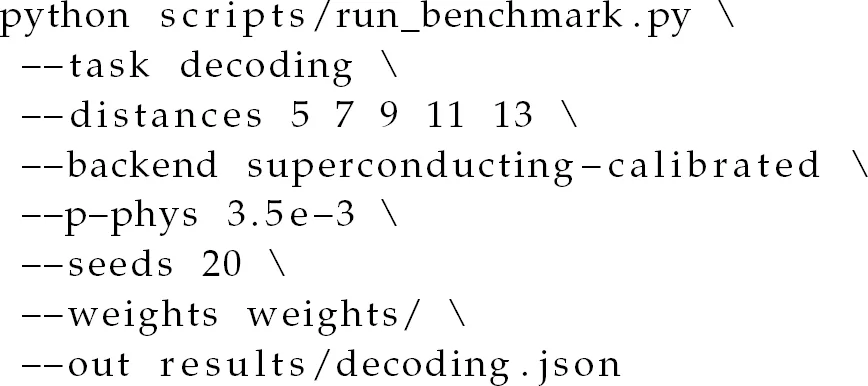

**Listing 4. VQE chemistry benchmark (figures 2–3 and tables 3–4).**

**Figure:**
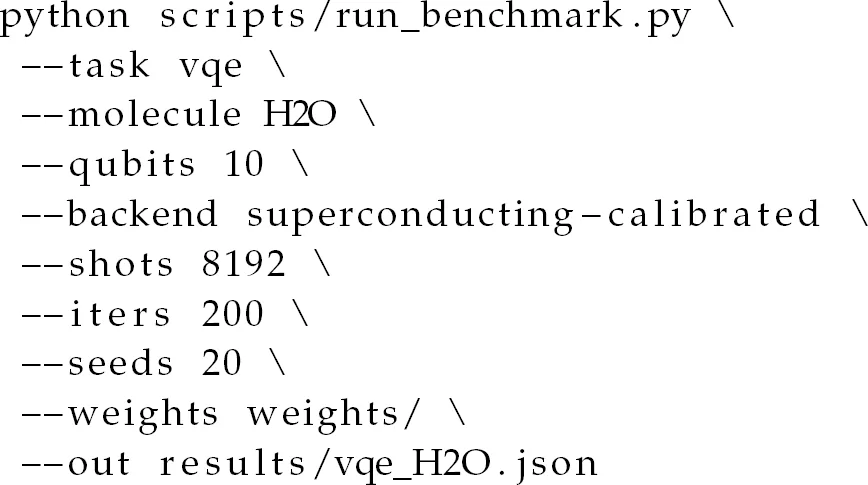

**Listing 5. Ablation study (table 2).**

**Figure:**
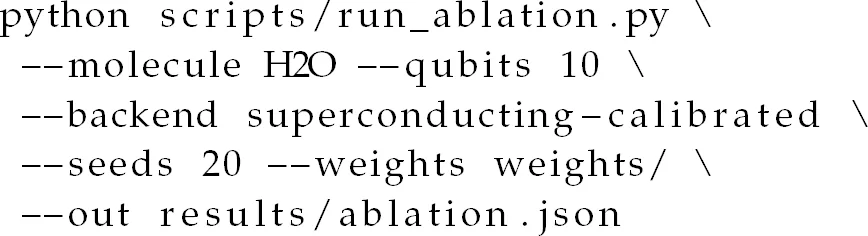

**Listing 6. Challenge proof-of-concept (table 4).**

**Figure:**
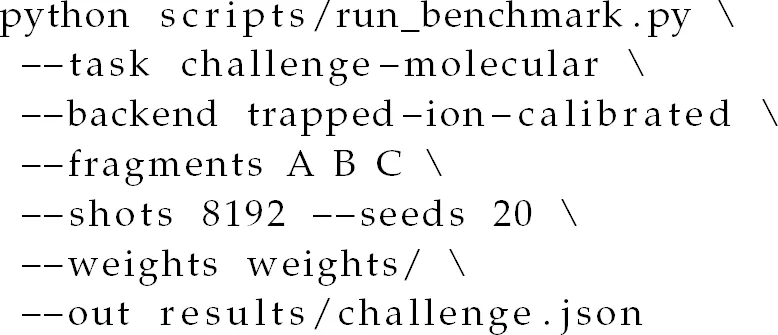

**Reference data and figure reproduction.** Raw benchmark JSON files are included in data/reference/. Run python scripts/plot_all.py –data data/reference/ to regenerate all figures using numpy and matplotlib.

**Device-calibrated noise models.** Noise parameters are derived from published characterization data [22,27,28] and loaded automatically via qcop.noise.load_calibrated(backend). All parameters and their sources are documented in configs/noise_models/.

## Data Availability

All code, trained model weights, reproduction scripts, benchmark configuration files, raw result JSON files and device-calibrated noise models are openly available. Source code and reproduction scripts (Apache 2.0 licence): http://github.com/hekimoghlu/QCOP. Trained model weights (SCE, HSD, OCL; CC-BY-4.0 licence): http://huggingface.co/thehekimoghlu/QCOP. Reference benchmark data and scripts to regenerate all figures/tables: included in the GitHub repository (data/reference/directory). The paper also includes a Zenodo DOI of itself http://zenodo.org/records/19674886. The GitHub repository contains complete installation instructions, benchmark scripts (e.g. run_benchmark.py, run_ablation.py) and verification commands to reproduce all results reported in the paper (*n* = 20 seeds, device-calibrated noise models from published characterization data). No proprietary software or hardware access is required beyond standard open-source quantum computing libraries (Qiskit Aer, pytket-qulacs). All materials are sufficient for full replication of the simulations and decoding benchmarks. Data and relevant code for this research work are stored in GitHub: http://github.com/hekimoghlu/qcop/ and have been archived within the Zenodo repository: http://doi.org/10.5281/zenodo.22044703.
